# Kinetics and Mechanism of In(III) Ions Electroreduction on Cyclically Renewable Liquid Silver Amalgam Film Electrode: Significance of the Active Complexes of In(III)—Acetazolamide

**DOI:** 10.3390/molecules28072942

**Published:** 2023-03-25

**Authors:** Agnieszka Nosal-Wiercińska, Marlena Martyna, Alicja Pawlak, Aleksandra Bazan-Woźniak, Robert Pietrzak, Selehatin Yilmaz, Sultan Yağmur Kabaş, Anna Szabelska

**Affiliations:** 1 Department of Analytical Chemistry, Institute of Chemical Sciences, Faculty of Chemistry, Maria Curie-Sklodowska University, Maria Curie-Sklodowska Sq. 3, 20-031 Lublin, Poland; 2 Faculty of Chemistry, Adam Mickiewicz University in Poznań, Uniwersytetu Poznańskiego 8, 61-614 Poznań, Poland; 3 Department of Chemistry, Faculty of Science and Arts, Çanakkale Onsekiz Mart University, Çanakkale 17020, Turkey; 4 Department of Chemistry and Chemical Processing Technology Programs, Program of Laboratory Technology, Lapseki Vocational School, Canakkale Onsekiz Mart University, Lapseki, Çanakkale 17020, Turkey; 5 Department of Prosthetic Dentistry, Medical University in Lublin, Karmelicka Str. 7, 20-093 Lublin, Poland

**Keywords:** R–AgLAFE electrode, In(III) ions electroreduction, active complexes, catalytic activity, kinetic parameters, thermodynamic parameters

## Abstract

The results of kinetic measurements revealed an accelerating effect of acetazolamide (ACT) on the multistep In(III) ions electroreduction in chlorates(VII) on a novel, cyclically renewable liquid silver amalgam film electrode (R–AgLAFE). The kinetic and thermodynamic parameters were determined by applying the DC polarography, square-wave (SWV) and cyclic voltammetry (CV), as well as electrochemical impedance spectroscopy (EIS). It was shown that ACT catalyzed the electrode reaction (“cap-pair” effect) by adsorbing on the surface of the R–AgLAFE electrode. The catalytic activity of ACT was explained as related to its ability to form active In(III)- acetazolamide complexes on the electrode surface, facilitating the electron transfer process. The active complexes constitute a substrate in the electroreduction process and their different structures and properties are responsible for differences in the catalytic activity. The determined values of the activation energy $\Delta H^{\neq }$ point to the catalytic activity of ACT in the In(III) ions electroreduction process in chlorates(VII). Analysis of the standard entropy values $\Delta S^{0}$ confirm changes in the dynamics of the electrode process.

## 1. Introduction

Indium, a heavy-metal element, is used, e.g., in the thin-film coatings of the liquid crystal display screens (LCDs), in CD/DVD players, computers, game consoles, solar cells and electroluminescent lamps [1]. High demand for indium has prompted development of methods for its recovery in the recycling process and the search for new and effective methods of its determination. So far, several low-cost, fast, selective, sensitive and highly reproducible methods have been proposed [2,3,4,5,6,7,8,9,10,11,12,13,14,15,16] for determination of indium.

In this study, the electrochemical reduction of In(III) ions was investigated in the presence of acetazolamide, whose impact changes the kinetics of this reaction. In fact, acetazolamide is a carbonic anhydrase drug inhibitor used as a diuretic as well as for the treatment of many diseases from glaucoma to epilepsy [17,18]. In this context, the studies of the mechanism and electroreduction kinetics seem to be justified as leading to deeper explanation of the drugs’ metabolic pathway or in vivo redox processes with their participation. The study is expected to contribute to explaining the mechanism of drugs’ action and control its release in the human body. All measurements were performed in the chlorates(VII) solutions on the novel, cyclically renewable liquid silver amalgam film electrode R–AgLAFE [19]. This electrode is clean, non-toxic and makes a perfect alternative to a dropping mercury electrode (DME). Moreover, it can be refreshed cyclically before each measurement. The weak complex-forming properties of ClO_4_^−^ ions, their the tendency to destruct the water structure and the fact that chlorate ions adsorb to a small extent on the mercury surface prompted the choice of chlorate(VII) solution as the basic electrolyte [20].

The experimental methods applied were DC polarography, square-wave voltammetry, cyclic voltammetry and electrochemical impedance spectroscopy. 

The information on the influence of organic molecules on the mechanisms of electrode processes is vital as it permits explanation of the reaction course and proposition of its technological and pharmacological applications. Organic molecules containing nitrogen or sulfur atoms with free electron couples, that may be bonded to or poorly adsorbed to the electrode surface in a wide range of potentials, may act as catalysts of the electrode processes, generally according to the “cap-pair” rule [21]. The mechanism of reduction of metal cations in the presence of a catalytic effect under the “cap–pair” conditions includes chemical reactions (formation of unstable complexes) and heterogenic charge transfer processes of the complexes which are electrochemically active on the electrode surface [22]. 

As the electrodeposition of indium on cyclically renewable liquid silver amalgam film electrode R–AgLAFE is a quasi-reversible process, it is possible to observe changes in the rate of this reaction under the influence of various adsorbates (both catalysts and inhibitors). 

ACT is adsorbed on a R–AgLAFE electrode and accelerates the reduction of In(III) ions in chlorates(VII) solutions. Simultaneously, it does not show electrochemical activity in the potential reduction range of the analyzed depolarizer.

Measurements were carried out on a cyclically renewable liquid silver amalgam film electrode [19] at electric potentials close to those at the border of cell membranes of living cells. Thus, the results of the study may be helpful to solve various scientific problems in biology, medicine and pharmacology in the processes taking place in the presence of acetazolamide.

Results of the studies are expected to bring a new perspective for the electrochemical determination of In(III) ions with acetazolamide because of its cleanliness, rapidity, low cost, selectivity, sensitivity and high reproducibility. “Cap-pair” effect opens an opportunity for the determination of metal ions in solutions with weak complexation properties [21].

## 2. Results and Discussion

**Figure 1:**
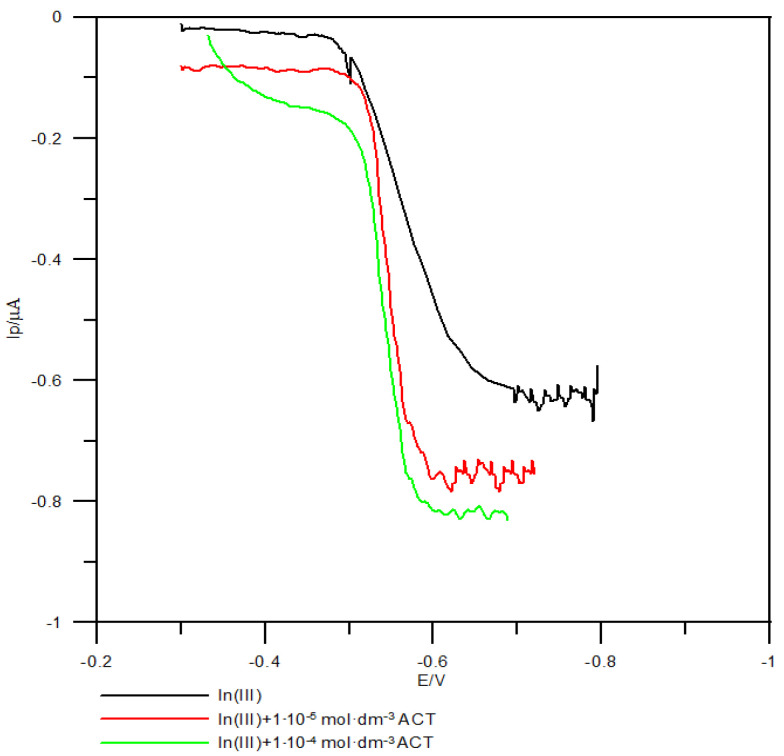
DC voltammograms of 1 × 10^−3^ mol·dm^−3^ In(III) electroreduction in 1 the mol·dm^−3^ chlorates(VII) solution in the acetazolamide presence. The concentrations of acetazolamide are given on the plot.

The addition of acetazolamide to the basic electrolyte solution containing 1 × 10^−3^ mol·dm^−3^ In(III) ions affects the magnitude of the limiting current (Figure 1). In contrast, a further increase in the concentration of ACT does not cause major changes in the limiting current. The slope of the polarographic wave increases, indicating a rise in the rate of the In(III) ions electroreduction process in the presence of the studied substance. However, over the concentration of 1 × 10^−4^ mol·dm^−3^ ACT, the slope of the wave decreases significantly. The electrode process becomes less reversible [22].

**Figure 2:**
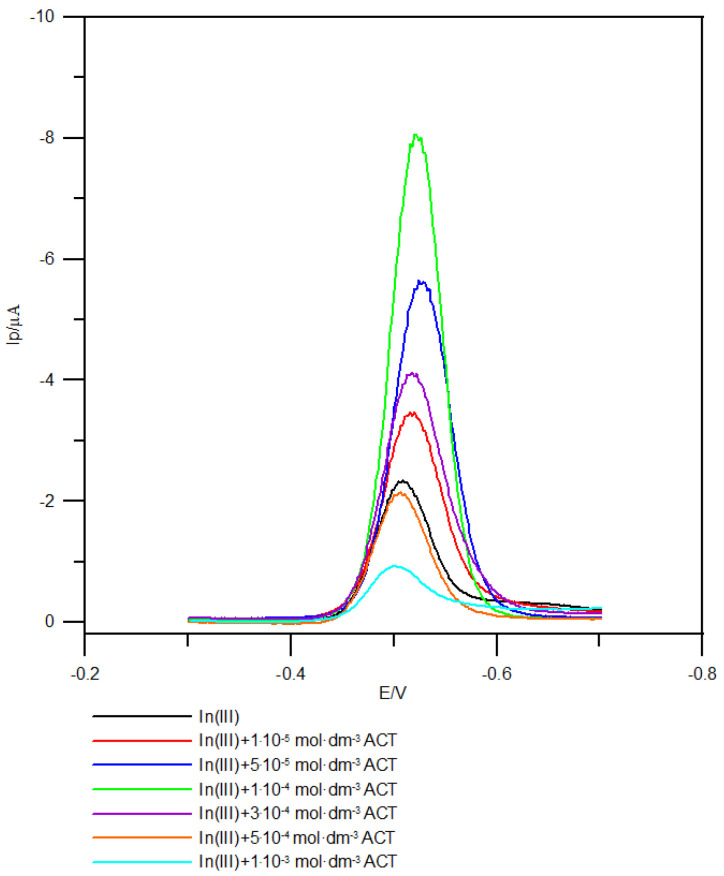
SWV voltammograms of 1 × 10^−3^ mol·dm^−3^ In(III) electroreduction in 1 mol·dm^−3^ chlorates(VII) solution in the acetazolamide presence. The concentrations of acetazolamide are given on the plot.

**Figure 3:**
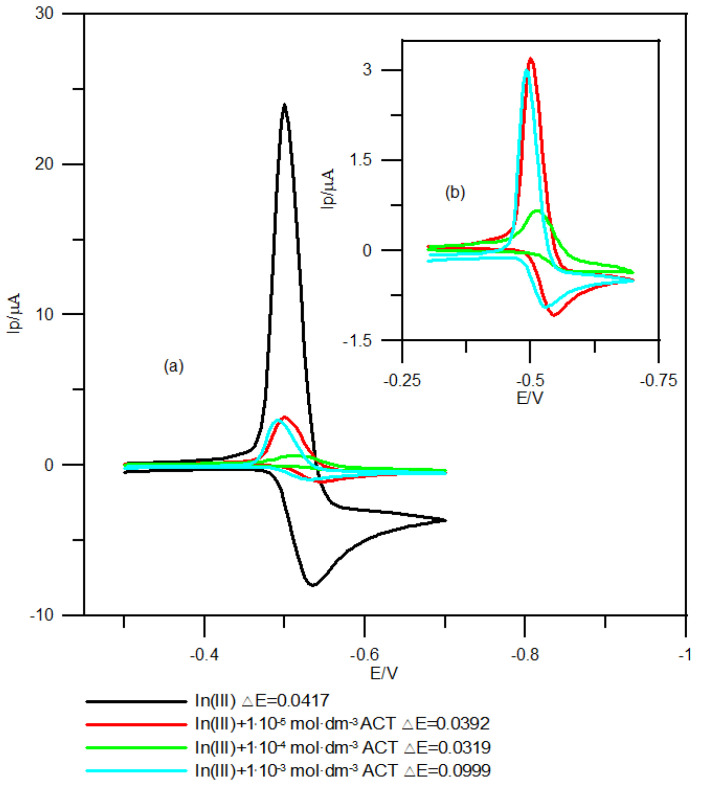
(**a**) Cyclic voltammograms of 1 × 10^−3^ mol·dm^−3^ In(III) electroreduction in the 1 mol·dm^−3^ chlorates(VII) solution in the acetazolamide presence. (**b**) Cyclic voltammograms of 1 × 10^−3^ mol·dm^−3^ In(III) electroreduction in the 1 mol·dm^−3^ chlorates(VII) solution in the presence of ACT without the basic electrolyte. The concentrations of acetazolamide, together with the corresponding peak potential separation values, are given on the plot. The scan rate was 50 mV s^−1^.

Similar changes in the reversibility of the In(III) ions electroreduction with increasing concentration of acetazolamide are indicated by the SWV and CV voltammograms (Figure 2 and Figure 3). As shown in Figure 2, both the addition and increase in the concentration of acetazolamide in the basic electrolyte solution result in an increase in the current of SWV peaks and also a decrease in their width at mid-height. These changes prove the increase in the reversibility of the In(III) ions electroreduction process. It is also noted that above the concentration of 1 × 10^−4^ mol·dm^−3^ ACT, the SWV peak currents become smaller.

The values of ΔE (obtained from the CV curves Figure 3a,b) decrease, compared to those obtained for the basic electrolyte (1 × 10^−3^ mol·dm^−3^ In(III) in 1 mol·dm^−3^ chlorates(VII)). Above the ACT concentration of 1 × 10^−4^ mol·dm^−3^, a definite increase in ∆E is observed, suggesting changes in the dynamics of the kinetics of the electroreduction process towards inhibition [22].

**Figure 4:**
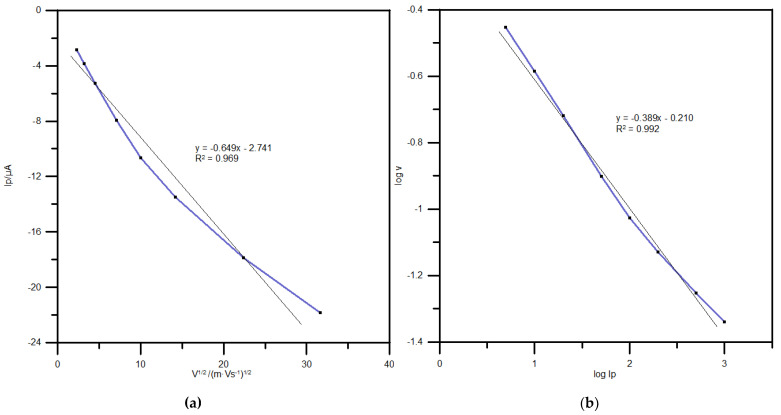
(**a**) Dependence of the cathodic peak current for the reduction of In(III) (*I*_p_) on the square root of the scan rate (*v*^1/2^). (**b**) The *log-log* dependence of the reduction peak current and the scan rate over the interval from 5 to 1000 mVs^−1^. Each point is the average of three measurements.

In order to highlight the differences between the mechanisms, cyclic voltammograms were recorded for a solution containing 1 × 10^−3^ mol·dm^−3^ In(III) using different scan rates (Table 1) and the resulting curves were analyzed for the dependence of the natural logarithm of the cathodic (ln|I_pc_|. Figure 4a,b) and anodic peak current as well as the potential of the cathodic peaks (E_pc_. Figure 4) on the natural logarithm of the scan rate *v*.

As illustrated in Figure 4a,b and Table 2, the increase in *v* results in increasing cathodic and anodic peak current. The correlation coefficient of peak current and square root of the scan rate is 0.985 (it was expected about 1.0) and the slope of the plot of the logarithm of peak current versus the logarithm of scan rate (close to 0.5) takes a value of 03893, indicating that the process is predominantly diffusion-controlled [23].

**Figure 5:**
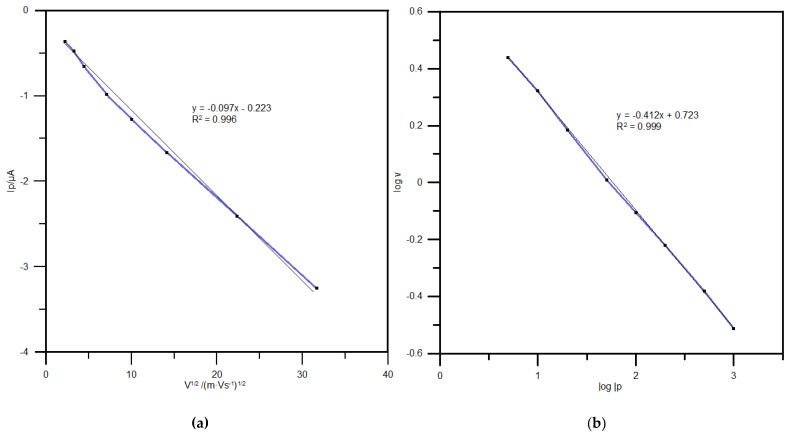
(**a**) Dependence of the cathodic peak current for the reduction of In(III) + 1 × 10^−4^ mol·dm^−3^ acetazolamide (*I*_p_) on the square root of the scan rate (*v*^1/2^). (**b**) The *log-log* dependence of the reduction peak current and the scan rate over the interval from 5 to 1000 mVs^−1^. Each point is the average of three measurements.

The shift in E_pc_ towards more negative potentials and the increase in the peak separation (ΔE. Table 2) with the rise in *v,* prove that the rate-determining step in the investigated electroreduction of In(III) ions is the charge transfer across the electrode–electrolyte interface. A similar procedure was applied while recording the cyclic voltammograms in the presence of ACT (Figure 5a,b).

The correlation coefficient of the peak current and square root of the scan rate is 0.9980 (it was expected to be about 1.0) and the slope of the plot of the logarithm of peak current versus the logarithm of scan rate (close to 0.5) is equal to 0.412. The above relations and the constancy of the cathodic peaks potential as well as smaller changes in ΔE for all tested concentrations of ACT indicate that the process is controlled by diffusion and is affected by adsorptive processes [23].

The cathodic peaks of In(III) ions electroreduction are always smaller than the anodic ones. This indicates that the process of In(III) ions electroreduction in 1 mol·dm^−3^ chlorates(VII) in the presence of ACT is controlled by the kinetics of the reaction preceding the passage of electrons [22].

**Figure 6:**
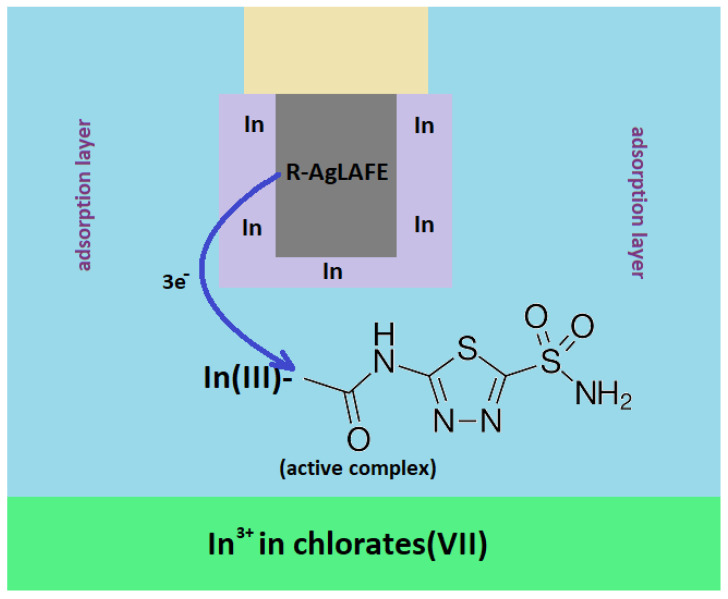
Scheme of In(III) ions electroreduction in chlorates(VII) including the mediating role of active complexes in the charge (electron) transfer.

Slight changes in the difference between the anodic peak potential and the cathodic peak potential Δ*E* due to changes in the polarization rate *v* (especially at low electrode polarization rates Table 1) confirm the control of 1 × 10^−3^ mol·dm^−3^ In(III) ions electroreduction in the presence of ACT by the preceding reaction, which probably consists in the formation of active complexes which mediate electron-transfer. The In-ACT complexes [22] are definitely localized inside the adsorption layer (Figure 6).

**Scheme 1:**
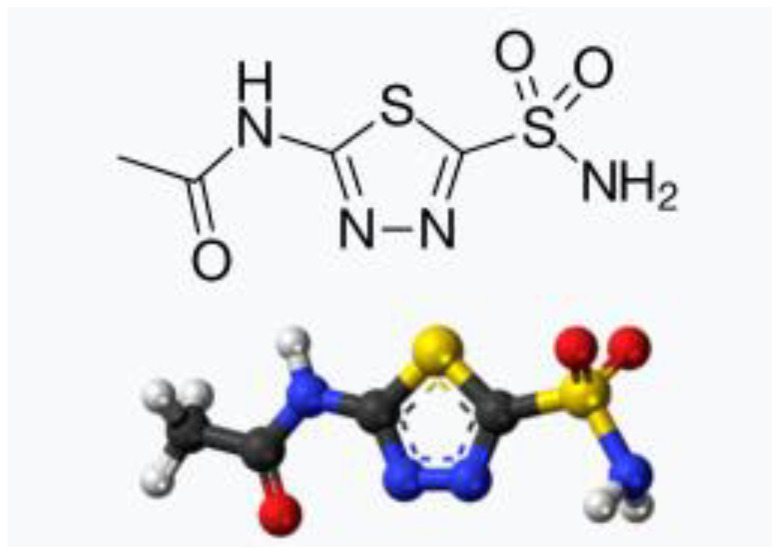
Scheme of acetazolamide structure.

As shown earlier, ACT is adsorbed on the electrode [24] and can shift the equilibrium complexation of In(III) ions favorably. It should also be noted that acetazolamide has complex-forming properties, most likely due to its structure containing free electron pairs at the sulfur and nitrogen atoms (Scheme 1).

The lack of marked changes in the value of the formal potential $E_{f}^{0}$ of In(III) ions electroreduction with increasing ACT concentration in the chlorates(VII) solutions (Table 3) proves the absence of stable In–ACT complexes in the supporting electrolyte solution under study [22]. Moreover, the obtained changes in $E_{f}^{0}$ also testify to the low stability of the complexes.

It should be emphasized, however, that the catalytic effect of ACT must be related to its ability to remove molecules of coordinated water from the inner hydration shell by establishing a bond to the indium central ion [22].

**Figure 7:**
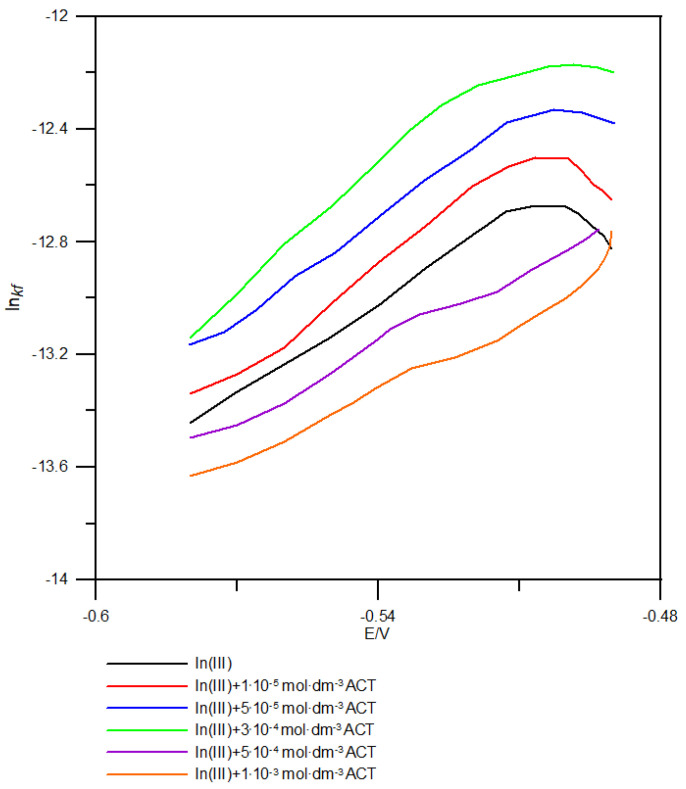
Dependence of the rate constants $k_{f\;}$ of 1 mol·dm^−3^ In(III) ions electroreduction in the 1 mol·dm^−3^ chlorates(VII) in the presence of acetazolamide on the electrode potential. The concentrations of acetazolamide are given on the plot.

It is also important to note the different character of the dependence $lnk_{f}=f\left(E\right)$ (Figure 7) above ACT concentration of 1 × 10^−4^ mol·dm^−3^, which may suggest changes in the electrode mechanism [24].

A similar effect was observed for In(III) in the presence of higher concentrations of thiourea and its selected derivatives [25] and for Bi(III) in the presence of amino acids [26] or for Zn(II) in the presence anionic surfactant sodium 1-decanesulfonate [27]. 

The acceleration effect of the In(III) ions electroreduction by acetazolamide (as theoretically inferred) is due to the lowering of the activation barrier. It is caused by a more efficient interpenetration of the orbital of In–acetazolamide complexes compared to the In(III) ions [28,29]. The acceptor orbital of the In(III) aqua complex ${\left[In{\left(H_{2}O\right)}_{6}\right]}^{3+}$ is probably significantly shifted toward the central atom. Thus, it can be assumed that the formation of the “surface” complex changes the structure of the acceptor orbital, facilitating the passage of electrons in the electroreduction process [28,29,30].

Moreover, the effect of the catalyst on the passage of the first electron is usually much greater than on the passage of the successive ones. This provides the evidence that the In(III) ions complexes with the accelerating substance are already formed before the passage of the first electron, which is the slowest stage and determines the speed of the entire process [22].

According to literature data, in the acidic non-complexing electrolyte solutions, the ${\left[In{\left(H_{2}O\right)}_{6}\right]}^{3+}$ ion has a very small rate of hydration loss. Consequently, the total electrode process consists also of chemical steps leading to labilization of ${\left[In{\left(H_{2}O\right)}_{6}\right]}^{3+}$ hydration envelope [28,29]. Based on the studies carried out by Nazmutdinov and coworkers, it can be concluded that in such solutions, the In(III) ions electroreduction follows the equation [29]:

$${\left[In{\left(H_{2}O\right)}_{6}\right]}^{3+}+e^{-}\to {\left[In{\left(H_{2}O\right)}_{6}\right]}^{2+}+e^{-}\to {\left[In{\left(H_{2}O\right)}_{6}\right]}^{+}+e^{-}\to {\left[In{\left(H_{2}O\right)}_{6}\right]}^{0}.$$

By analogy with the mechanism proposed by Nazmutdinov, the mechanism of the chemical stage of the above mentioned reaction of the active complexes formation on the surface of the electrode facilitates the reaction of In(III) ions electroreduction. The chemical stage is probably related to the partial loss of the hydration envelope by In(III) ions which, locating close to OHP, change their electrostatic potential.

The effect of temperature on the rate of the studied electroreduction processes was also demonstrated. The determined values of the activation energy $\Delta H^{\neq }$ [30,31] confirmed the catalytic activity of ACT on the In(III) ions electroreduction process in chlorates(VII). It should be pointed out that above the ACT concentration of 1 × 10^−4^ mol × dm^−3^, the values of the activation energy $\Delta H^{\neq }$ increase, which confirms a change in the dynamics of acceleration of the electroreduction process towards inhibition (Table 4).

The changes in the standard entropy value $\Delta S^{0}$ of the In(III) ions electroreduction process in the presence of acetazolamide in the 1 mol·dm^−3^ chlorates(VII) (Table 4) also suggest those in the dynamics of the electrode process. The significant changes in $\Delta S^{0}$ above the concentration of 1 × 10^−4^ mol·dm^−3^ ACT may indicate changes in the electrode mechanism, as mentioned previously [30].

Changes in the mechanism are usually related to changes in the kinetics of the electrode process. A significantly higher concentration of ACT can affect the reorganization of the adsorbed molecules on the electrode—for example, from a flat arrangement to a more vertical one, or vice versa—which can be associated with the impeded access of the depolarizer to the electrode surface. However, it should be noted that adsorption of the catalyst—as a necessary condition for accelerating the process of In(III) ions electroreduction—does not determine the magnitude of the catalytic effect. Based on the parameters of cyclic voltammetry curves, the values of transition coefficients α and the standard rate constants $k_{s}$ of In(III) ions electroreduction and in the presence of acetazolamide were determined. The calculated kinetic parameters indicated the catalytic effect of acetazolamide and its magnitude (Table 5).

The increase in the value of the transition coefficients (Table 5) after introducing acetazolamide into the electrolyte solution indicates an increase in the reversibility of the In(III) ions electroreduction process. Above the concentration of 1 × 10^−4^ mol·dm^−3^ ACT, the α values decrease slightly, indicating changes in the reversibility of the electrode process toward inhibition. This translates also into an increase in the standard rate constants $k_{s}$ (Table 5) which confirms the influence of ACT on the kinetics of the In(III) ions electroreduction process.

## 3. Materials and Methods

### 3.1. Chemicals

The solutions used in the studies of the following reagents of analytical grade— NaClO_4_ and HClO_4_ and acetazolamide (Sigma-Aldrich, Missouri, USA) in redistilled water—were purified with the Millipore Milli-Q system. The supporting electrolyte was made dissolving In(NO_3_)_3_ in HClO_4_. The resulting solution was quantitatively transferred to a flask which was filled up to the mark with the solution of 1 mol·dm^−3^ NaClO_4_. The concentration of In(III) ions in the studied solution was always the same and was 1 × 10^−3^ mol·dm^−3^. During the experiments, acetazolamide solutions were used in a range of concentrations from 5 × 10^−5^ to 1 × 10^−3^ mol·dm^−3^. All solutions were freshly prepared just before the measurements and deaerated using the purge of nitrogen which was passed over the solution during the measurements.

### 3.2. Apparatus

**Figure 8:**
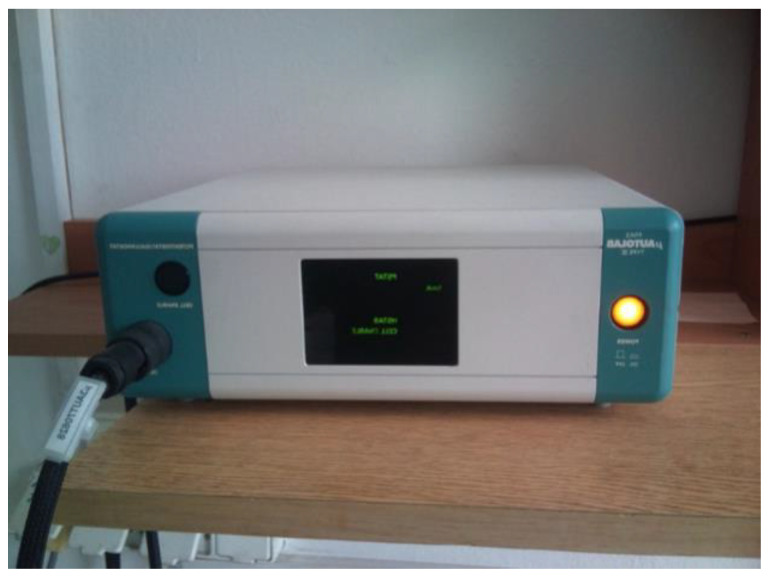
Potentiostat/galvanostat—μAutolab GpES.

The experiments were performed in a thermostated vessel using the electrochemical analyzer μAutolab Fra 2/GPES (Eco Chemie, Utrecht, The Netherlands) frequency response analyzer (Figure 8). To determine the thermodynamic parameters, measurements were made at the following temperatures: 288, 293, 298 and 303 ± 0.1 K.

**Figure 9:**
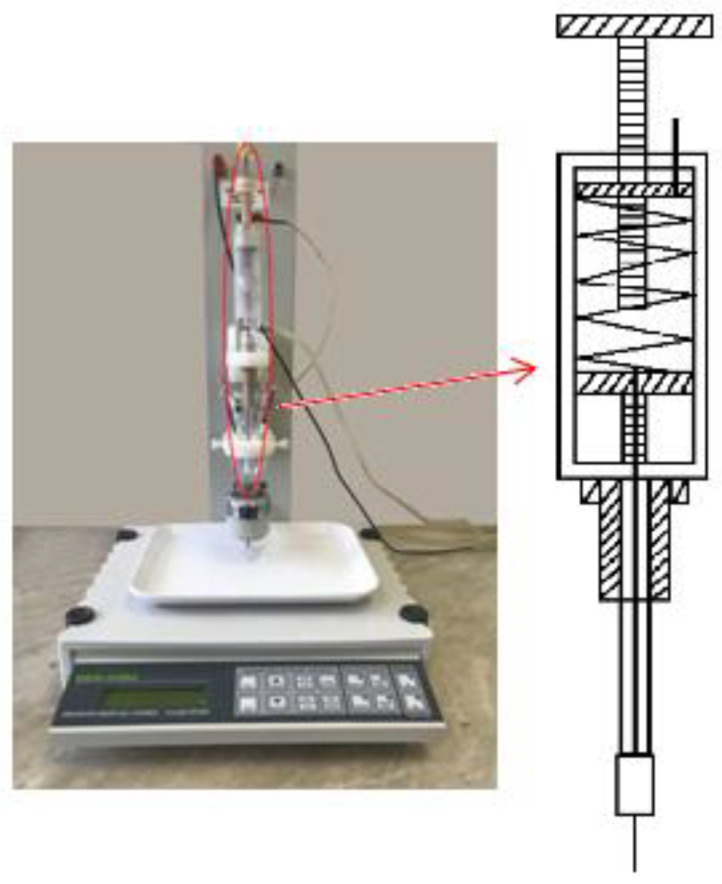
Three-electrode voltammetric cell on the automatic stand with the centrally fixed working electrode (R–AgLAFE).

A three-electrode system was placed in the tripod and the arrangement of the electrodes was as follows:

- The Ag/AgCl/3M KCl electrode as a reference;
- A platinum wire as an auxiliary electrode;
- A cyclically renewable liquid silver amalgam film electrode (R–AgLAFE) with the surface area of 17.25 mm^2^ as the working electrode (Figure 9).

The construction of the cyclic renewable liquid silver amalgam film R-AgLAFE electrode [19] permits a smooth, precise change in the contact surface of the working electrode with the tested environment, automatic regeneration of the layer of liquid silver amalgam without contact with atmospheric air and control of the time of movement of the working electrode from the sensor housing the tested solution, with the preservation of its properties acquired during regeneration. In addition, the method of applying and homogenizing the layer of liquid amalgam and moving the working electrode between the solution and the amalgamation chamber by means of a linear pneumatic actuator ensures cyclic reproducibility and reproducibility of the surface, excellent homogeneity and uniformity of the film and constancy of the applied film layer density.

Owing to all the above properties, the proposed electrode makes an alternative to the mercury drop electrode as it guarantees similar quality and performance parameters as those of HMDE. It also fits in with the theme of green chemistry as it permits a significant reduction in the use of toxic mercury during the manufacture of the amalgam film. Moreover, and most importantly, it provides the opportunity to study electrode processes under the “cap-pair” conditions [21].

### 3.3. Measurement Procedures

#### Kinetics and Thermodynamic Procedure

The study of the kinetics and mechanism of the electrode process entailed the necessity of determining the kinetic and thermodynamic parameters.

The values of diffusion coefficients (*D_ox_*) necessary for the calculation of kinetic parameters of the In(III) ions electroreduction in the studied solutions were determined based on the Ilkovič equation for the boundary current controlled by diffusion [32].

In practice, the diffusion coefficient is determined in a simpler way using the comparative method. Finally, the following formula is used to calculate *D_ox_* [32]:

(1) $$D_{ox*}^{1/2}={\left(\frac{I_{dl*\;}^{*}x\;\sqrt{D_{ox}}}{I_{dl}}\right)}^{2}$$

The details are described elsewhere [32].

The values of formal potential ($E_{f}^{0}$), transfer coefficient (α) and standard rate constants ($k_{s}$) were determined as in [30].

The activation polarization resistances ($R_{A}$) were determined using the electrochemical impedance spectroscopy for $E_{f}^{0}$ and from the dependence $Z^{\prime }=f\left(\omega Z^{\prime \prime }\right)$ or $Z^{\prime }=f\left(Z^{\prime \prime }\right)$ [31] where $Z^{\prime }$ is the real part and $Z^{\prime \prime }$ is the imaginary part of the cell impedance.

**Scheme 2:**
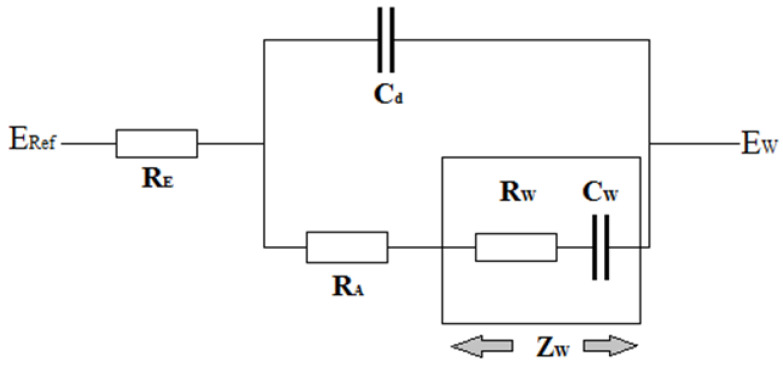
Randles’ electric al equivalent circuit diagram [33]. $E_{\mathrm{Ref}}$—reference electrode; $E_{W}$ —working electrode; $R_{E}$ —resistance of electrolyte, electrode and bonds; $C_{d}$ —differential capacity of the double layer; $R_{A}$ —activation resistance;$\;Z_{W}$ —Wartburg impedance; $R_{W}$ —Wartburg resistance; $C_{W}$ —Wartburg capacity.

In order to quantitatively interpret the experimental results obtained, the impedance of the system under study was described using the corresponding equivalent circuit proposed by Randles (Scheme 2) [33].

From the charge transfer resistance values [30] as a function of DC potential, the values of the apparent rate constant (*k_f_*) of the In (III) electroreduction in the studied systems were obtained:

(2) $$R_{ct}=\frac{RT}{n^{2}F^{2}c_{0}k_{f}S}\cdot \frac{a_{0}/k_{f}+1+r_{s\;}exp\left(b\right)}{\alpha a_{0}/k_{f}+r_{s}exp\left(b\right)}$$

The details are described elsewhere [30].

The enthalpies of activation $\left(\Delta H^{\neq }\right)$ for the In(III) electroreduction in the studied systems were determined according to equation [30]:

(3) $$\Delta H^{\neq }=R\frac{dlnk_{s}}{d\frac{1}{T}}$$

whereas the standard reaction entropy $\left(\Delta S^{0}\right)$ [30]:

(4) $$\Delta S_{Bi\left(III\right)/Bi\left(Hg\right)}^{0}=2F\frac{dE_{f}^{0}}{dT}$$

### 3.4. Experimental Operating Conditions

In the voltammetric or polarographic measurements, the optimal experimental operating conditions were as follows: the scan rate 2 mV s^−1^ for DC; the scan rate 5–1000 mV s^−1^ for the cyclic voltammetry; the step potential 2 mV; the pulse amplitude 20 mV; the frequency 120 Hz for the square wave voltammetry. No fewer than three scans were performed for each measurement. The range of the tested potentials was constantly changed to study the variety of processes that can occur. The impedance experiments were performed in the frequency range from 50 to 50,000 Hz with the sinusoidal signal of 10 mV amplitude at the open circuit potential (OCP).

## 4. Conclusions

The catalytic ability of acetazolamide can be explained by the formation of the above-mentioned active complexes on the electrode surface between the ACT molecules adsorbed on it and the indium aqua complex (“cap-pair” effect). The electrode surface is the optimal area for the formation of such unstable and charge exchange-mediated complexes as a significant local ACT concentration is a result of adsorption of ACT on the R–AgLAFE electrode.

These complexes are the substrate in the electroreduction process. The stages of dehydration and formation of active complexes are much faster than the stages of electron transition, which makes it impossible to detect these chemical steps. A probable consequence of increasing ATC concentration (above 1 × 10^−4^ mol·dm^−3^) is a change in the arrangement of the molecules absorbed on the electrode. As a result, the access of depolarizer to the electrode surface is difficult and the electrode process is inhibited by ACT (with increasing ACT concentration, a decrease in the SWV peak currents and an increase in the distance between the anode and cathode peaks on the CV voltammograms are observed). It was shown that the magnitude of the catalytic effect is mainly related to the equilibrium reaction of the formation of In–ACT active complexes before the passage of subsequent electrons.

## Figures and Tables

**Table 1:** Changes in ∆E of the 1 × 10^−3^ mol·dm^−3^ In(III) ions electroreduction process and in the presence of acetazolamide in 1 mol·dm^−3^ of chlorates(VII) at the rate of polarization v.

10^3^ C_In(III)_ + 10^4^ C_ACT_ /mol∙dm^−3^					∆E/V			
					v/mV·s^−1^			
		5	10	20	50	100	200	500
**0.00**	0.0292	0.0313	0.0324	0.0333	0.0417	0.0504	0.0625	0.0645
**0.10**	0.0260	0.0265	0.0273	0.0321	0.0392	0.0468	0.0571	0.0592
**0.30**	0.0230	0.0232	0.0238	0.0310	0.0368	0.0440	0.0520	0.0558
**0.50**	0.0222	0.0226	0.0230	0.0297	0.0343	0.0381	0.0457	0.0501
**1.00**	0.0207	0.0211	0.0218	0.0281	0.0319	0.0340	0.0398	0.0472
**3.00**	0.0541	0.0547	0.0555	0.0613	0.0648	0.0710	0.0768	0.0910
**5.00**	0.0602	0.0608	0.0614	0.0690	0.0789	0.1141	0.1268	0.1373
**10.0**	0.0758	0.0762	0.0767	0.0878	0.0999	0.1257	0.1358	0.1411

**Table 2:** The values of the slope of the dependencies of ln(|*I_p_*|) vs. ln(*v*), where *I_p_*—the peak current, *v*—the scan rate.

10^3^ C_In(III)_+ 10^4^ C_ACT_ /mol∙dm^−3^	Slope	
	*Cathodic*	*Anodic*
**0.00**	0.389 ± 0.029	0.485 ± 0.037
**0.10**	0.354 ± 0.027	0.500 ± 0.038
**0.50**	0.347 ± 0.027	0.479 ± 0.037
**1.00**	0.412 ± 0.031	0.479 ± 0.033
**3.00**	0.302 ± 0.023	0.483 ± 0.037
**5.00**	0.351 ± 0.027	0.431 ± 0.033

**Table 3:** Values of formal electroreduction potentials ($E_{f}^{0}$ ) of 1 × 10^−3^ mol·dm^−3^ In(III) ions in the 1 mol·dm^−3^ chlorates(VII) and the presence of acetazolamide.

10^3^ C_In(III)_ + 10^4^ C_ACT_ /mol∙dm^−3^	$E_{f}^{0}/V$
**0.00**	0.520
**0.10**	0.540
**0.30**	0.530
**0.50**	0.550
**1.00**	0.540
**3.00**	0.560
**5.00**	0.560
**10.0**	0.580

**Table 4:** The values of standard activation energy (Δ𝐻^#^) and standard reaction entropy (Δ𝑆^0^) for the In(III) ions electroreduction in the 1 mol·dm^−3^ chlorates(VII) and in the presence of 1 × 10^−3^ mol·dm^−3^ acetazolamide.

10^3^ C_In(III)_+ 10^4^ C_ACT_ /mol∙dm^−3^	$\Delta H^{\neq }/\mathrm{kJ}\cdot {\mathrm{mol}}^{-1}$	$\Delta {S^{0}}_{\mathrm{In}(\mathrm{III})/\mathrm{In}(\mathrm{Ag}-\mathrm{LAFE})}/\;\mathrm{kJ}\cdot {\mathrm{mol}}^{-1}$
**0.00**	36	430
**0.10**	21.49	378
**0.30**	19.64	352
**0.50**	16.26	339
**1.00**	11.87	325
**3.00**	29.04	335
**5.00**	38.18	351
**10.0**	45.67	397

**Table 5:** The values of cathodic transition coefficients (α), standard rate constants determined by the voltammetry ($k_{s}$) and impedance ($k_{f}$) methods of 1 × 10^−3^ mol·dm^−3^ In(III) ions electroreduction in 1 mol·dm^−3^ chlorates(VII) and in the presence of acetazolamide.

10^3^ C_In(III)_+ 10^4^ C_ACT_ /mol∙dm^−3^	α	k_s_ 10^4^/cm·s^−1^	
		CV	EIS
**0.00**	0.40	0.53	0.35
**0.10**	0.44	0.89	1.03
**0.30**	0.48	1.23	1.55
**0.50**	0.51	3.29	4.33
**1.00**	0.59	6.23	7.12
**3.00**	0.49	6.17	6.96
**5.00**	0.35	5.93	6.48
**10.0**	0.27	5.72	6.23

## Data Availability

https://www.mdpi.com/ethics, (accessed on 24 February 2023).
